# Air Pollution and Racial Disparities in Pregnancy Outcomes in the United States: A Systematic Review

**DOI:** 10.1007/s40615-023-01539-z

**Published:** 2023-03-10

**Authors:** Bonaventure S. Dzekem, Briseis Aschebrook-Kilfoy, Christopher O. Olopade

**Affiliations:** 1 Biological Sciences Division, Department of Medicine, The University of Chicago, Chicago, IL USA; 2 Center for Global Health, Biological Science Division, The University of Chicago, 5841 S Maryland Ave, suite G-120, Chicago, IL 60637 USA; 3 Internal Medicine Residency Program, Department of Medicine, The University of Chicago, Chicago, IL USA; 4 Department of Public Health Sciences, The University of Chicago, Chicago, IL USA

**Keywords:** Air pollution, Race, Ethnicity, Racial disparities, Pregnancy outcomes, Public health policy

## Abstract

**Background:**

Exposure to air pollutants and other environmental factors increases the risk of adverse pregnancy outcomes. There is growing evidence that adverse outcomes related to air pollution disproportionately affect racial and ethnic minorities. The objective of this paper is to explore the importance of race as a risk factor for air pollution-related poor pregnancy outcomes.

**Methods:**

Studies investigating the effects of exposure to air pollution on pregnancy outcomes by race were reviewed. A manual search was conducted to identify missing studies. Studies that did not compare pregnancy outcomes among two or more racial groups were excluded. Pregnancy outcomes included preterm births, small for gestational age, low birth weight, and stillbirths.

**Results:**

A total of 124 articles explored race and air pollution as risk factors for poor pregnancy outcome. Thirteen percent of these (*n*=16) specifically compared pregnancy outcomes among two or more racial groups. Findings across all reviewed articles showed more adverse pregnancy outcomes (preterm birth, small for gestational age, low birth weight, and stillbirths) related to exposure to air pollution among Blacks and Hispanics than among non-Hispanic Whites.

**Conclusion:**

Evidence support our general understanding of the impact of air pollution on birth outcomes and, specifically, of disparities in exposure to air pollution and birth outcomes for infants born to Black and Hispanic mothers. The factors driving these disparities are multifactorial, mostly social, and economic factors. Reducing or eliminating these disparities require interventions at individual, community, state, and national level.

## Introduction

According to the World Health Organization (WHO), 91% of the world’s population lives in places where air pollution exceeds recommended limits [1]. Globally, about 4.2 million deaths are attributed to exposure to ambient air pollution and an additional 3.8 million deaths to exposure to household air pollution, with a disproportionate burden among women and children [2, 3].

There is evidence that exposure to air pollution adversely affects human health [4–8]. Additionally, in the last two decades, evidence has emerged that exposure to a variety of pollutants in the air (carbon monoxide, nitrogen oxides, particulate matter, sulfur dioxide, etc.) and other environmental factors increase the risk of adverse pregnancy outcomes [9–12]. Further evidence is accumulating that adverse pregnancy outcomes such as low birth weight (LBW), preterm birth (PTB) and small for gestational age (SGA), maternal and infant mortality are more common in racial minority groups [13–15]. These pregnancy outcomes have been shown to have serious health consequences during the neonatal period and infancy [16–18], childhood [19–21], and adulthood [22–24]. Public health studies in the USA often incorporate race and ethnicity as factors to adjust for. Alternatively, results are often reported stratified by race/ethnicity. The adverse impact of air pollution in racial and ethnic minorities is attributed to factors including age, geographical location, socioeconomic status, and education [25–27]. Although this accumulating research offers solid evidence that exposure to air pollution increases the risk of adverse pregnancy outcomes in general, there is still a need to assess its specific impact on pregnancy outcomes in minority populations, considering growing racial disparities.

Here, we reviewed the epidemiologic studies investigating the effects of exposure to air pollution on pregnancy outcomes in the USA with results stratified by race and/or ethnicity to provide a comprehensive view of exiting research, including types of measurement and study designs, to inform future research directions and to deepen insight into this important topic.

## Methods

**Fig. 1:**
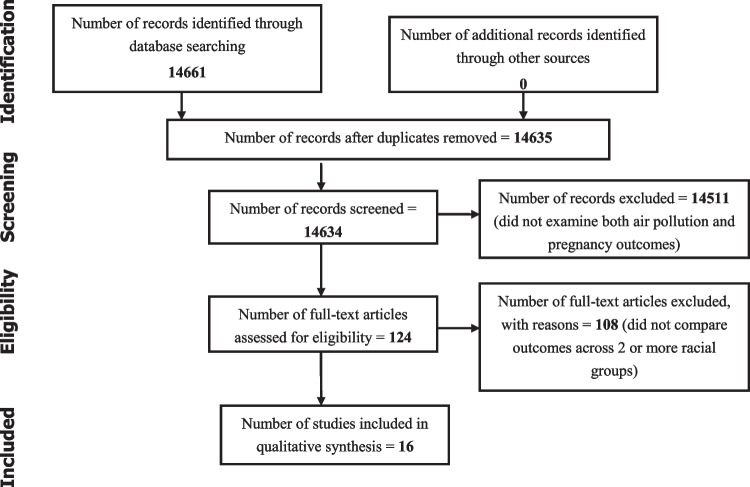
PRISMA flow diagram

The study process followed the recommendations of the PRISMA checklist for reporting systemic reviews and meta-analysis, where registration of the protocol is not mandated [28]. We searched the PubMed database for eligible studies that explored the effects of pregnancy outcomes on women across all races by combining Medical Subject Headings (MeSH). Key search terms used for the search included a combination of “air pollution” and one or more of the following: “pregnancy outcomes,” “birth outcomes,” “complications of pregnancy,” “race,” “ethnicity,” “black,” “white,” “African American,” and “continental population groups.” We included studies, published in English language, that explored air pollution and racial disparities in pregnancy outcomes and excluded articles that did not have racial comparisons and studies that did not specifically explore the effects of air pollution. We also excluded non-original reports (reviews, letters to the editor, commentaries), articles with unavailable full texts, and duplicated records. Searches with the above terms yielded 14,661 articles. The primary author filtered for studies in the English language, followed by filtering for studies that examined effects of air pollution on pregnancy outcomes and retained 124 eligible articles. Titles and abstracts of all retained studies were then reviewed for eligibility criteria by two reviewers. Any conflicts were resolved through a discussion between the authors. Full articles for eligible studies were accessed via journal sites. Two authors developed the data extraction sheet using Microsoft Excel. Data extraction was performed by the primary reviewer and was cross-checked by two reviewers to ensure accuracy. All discrepancies were resolved via a discussion between the authors. Quality assessment was done by two reviewers who evaluated risk for bias in the included studies using the National Institute of Health (NIH) risk tool [29]. Due to wide variations in methods of exposure capture, a meta-analysis was not performed. Of the 124 studies reviewed, 16 compared pregnancy outcomes among two or more racial groups. For each, we report the study design, sample size, type of pollutant, and method used to capture exposure. We also report the type of adverse pregnancy outcome, specifically, preterm births (PTB), small for gestational age (SGA), low birth weight (LBW), and stillbirths. We compared findings across non-Hispanic white, Hispanic, and Black race/ethnicity (Fig. 1).

## Results

All 16 studies that met review criteria were conducted between 1990 and 2014. Of these, 15 were retrospective cohort studies using birth registries and one was a case control study (Table 1). There was a considerable representation of states across regions of the USA. Sample sizes ranged from 1761 to 1,548,904 births. Different methods were used to estimate levels of air pollution exposure, including Bayesian measurement, Community Multi-scale Air Quality (CMAQ) model, data from United States Environmental Protection Agency (EPA), installation of ambient air monitors, proximity to major roads, and mixed methods. While most studies looked at a single pregnancy outcome, others considered multiple outcomes. Specific details of racial disparities in pregnancy outcomes across race are described below.

**Table 1:** Air pollution and racial disparities in pregnancy outcomes in the USA

Reference	Study design	Study years	Country	Study population/sample size	Total black	Total white	Total Hispanic	Method used to capture exposure	Outcome measure	Adverse pregnancy outcome Total	Adverse outcome Blacks	Adverse outcome Whites	Adverse outcome Hispanics
Benmarhnia T et al	Retrospective cohort	2005–2010	California USA	1,066,783	175,297	891,486	Not specified	Measurement of PM2.5 and NO2 by EPA	PTB, SGA	Prevalence PTB=10.9%, SGA=2.2%	PTB=15%; SGA=18.3%	PTB=11.3%; SGA=9.9%	Not specified
Gray SC et al	Retrospective cohort	2001–2006	North Carolina USA	457,642	22.8%	62.4%	14.8%	Bayesian measurement of PM2.5 and O3	LBW, SGA, PTB	LBW=7.3%; SGA=10.2%; PTB=9.8%	OR LBW=2.13(2.05,2.22); PTB=1.46(1.42,1.50)	Reference	LBW=0.99 (0.93, 1.05); PTB=0.78 (0.75, 0.81)
Wallace ME et al	Retrospective cohort study	2000–2008	Multi national	223,375	50,255	110,541	38,811	CMAQ model	PROM	PROM=7%	PROM=7.6%	PROM=6.9%	PROM=6.4%
Hao H, et al	Retrospective cohort	2002–2006	Georgia USA	511,658	161,583	329,152	Not Specified	CMAQ model CO, SO2, O3, NO2, PM2.5	PTB	PTB=9.3%	PTB=20,283	PTB=25,514	Not specified
Woodruff TJ et al	Retrospective cohort	1998–1999	California USA	4,098,740	15.0%	61.2%	18.8%	U.S. EPA ambient air quality measurement PM10, O3, CO, NO2, SO2	SGA, PTB	SGA=9.0%, PTB=9.9%	AOR for SGA=2.0 OR for PTB-1.9	Reference	Not specified
Kingsley SL et al	Retrospective cohort	2002–2012	Rhode Island USA	61,640	4,706	36,510	Not specified	Land-use regression and satellite remote sensing. PM2.5, BC	PTB		PTB=478	PTB=2,853	Not specified
DeFranco E et al	Retrospective cohort	2006–2010	Ohio USA	351,036	46.6%	44.8%	5.8%	U.S. EPA local measurement of PM2.5	Stillbirths	Stillbirths=0.5%	Stillbirth=8.6 per 1000	Stillbirth=3.7 per 1000	Stillbirth=5.7 per 1000
Nobles CJ et al	Retrospective cohort	2002–2010	Maryland USA	109,126	216	43,055	5,328	CMAQ model, SO2, NO2, PM10	FGR	FGR=1.5% (825)	FGR=8	FGR=708	FGR=88
Le HQ et al	Retrospective cohort	1990–2001	Michigan USA	164,905	93,078	68,164	Not specified	Ambient air monitors, SO2, O3CO, NO2, , PM10	PTB, SGA	nSGA=13,754 nPTB=24,954	SGA=69.1%, PTB=71.1%	SGA=28.6%, PTB=27.1%	
Miranda ML et al	Retrospective cohort	2004–2008	North Carolina USA	468,517	23.3%	60.3%	16.4%	Proximity to major road	LBW, PTB, SGA	LBW=6.8%, PTB=10.5%, 10.2%	LBW=11.6%, PTB=14.6%, SGA=16.1%	LBW=5.5%, PTB=9.1%, SGA=8.1%	LBW=5.0% PTB=9.9% SGA=9.3%
Laurent O et al	Retrospective cohort	1997–2006	California USA	70,000	6,261	30,548	23,678	Mixed method NOx, CO, O3, PM	LBW		LBW=3.19%	LBW=1.23%	LBW=1.59%
Bell ML et al	Retrospective cohort	1999–2002	California USA	358,504	10.7%	83.4%	Not specified	US EPA measurements on NO2, SO2, PM, CO	LBW	LBW=4.01%	LBW=−97.8 (−102.9 to −92.7)	Reference	Not specified
Rich DQ et al	Retrospective cohort	1999–2003	New Jersey USA	350,107	38,978	84,747	54,473	US EPA measurements PM2.5, NO2, SO2, CO	SGA	SGA=8%, VSGA=2%	SGA=24%	SGA=46%	SGA=30%
Geer LA et al	Retrospective cohort	1998–2004	Texas USA	1,548,904	10.6%	34.7%	50.7%	US EPA measurements PM, SO2 NO2, CO	LBW	LBW=2.8%	LBW=−168.1 (−170 to −165)	Reference	LBW=−64.1 (−66.0 to −62.3)
Ghosh JK et al	Case control	2003	CaliforniaUSA	1,761	7%	10.9%	73.6%	Air quality monitoring data	LBW		LBW=3.85 (1.54,9.79)	Reference	LBW=2.75 (1.32,5.72)
Kahr MK et al	Retrospective chohort	2011–2014	Texas USA	9004	10.7%	30.5%	69.5%	People commuting to work	PTB	PTB=10%	PTB=11%	PTB=32%	PTB=68%

### Air Pollution and Preterm Birth

Preterm birth was assessed in eight out of sixteen of the studies reviewed. Benmarhnia et al. [30] used decomposition analysis to understand the racial disparities in PTB in California. Two pollutants were included fine-grained particulate matter (PM_2.5_) and nitrogen dioxide (NO_2_). A higher prevalence of PTB was observed for non-Hispanic Black (NHB) mothers when compared with non-Hispanic White mothers. The predicted difference in probability of PTB between Black and White infants was 0.056 (95% CI: 0.054, 0.058). All included predictors explained 37.8% of the Black–White disparity. Overall, individual variables (17.5% for PTB) such as age and level of education, and neighborhood-level variables (16.1% for PTB) such as socioeconomic environment explained a greater proportion of the Black–White difference in birth outcomes than air pollution (5.7% for PTB).

In a cohort of 457,642 births in North Carolina, using Bayesian measurement of PM_2.5_ and ozone (O_3_), Gray et al. [27] reported that NHB and Hispanic mothers were more likely to have infants born with lower birth weight when compared with NHW mothers (−188.2 g, 95% CI: −184.3 to −192.1 and −47.3 g, 95% CI: −42.4 to −52.3, respectively).

Using the CMAQ model and measurements from stationary monitors, Hao et al. [31] investigated the association between 11 ambient air pollutants and the risk of PTB in the state of Georgia. They observed that all traffic-related pollutants (carbon monoxide (CO), NO_2_ PM_2.5_, elemental carbon) were associated with PTB (e.g., odds ratios for interquartile range increases in CO during the first, second, and third trimesters and total pregnancy were 1.005 (95% CI: 1.001, 1.009), 1.007 (95% CI: 1.002, 1.011), 1.010 (95% CI: 1.006, 1.014), and 1.011 (95% CI: 1.006, 1.017), respectively). Associations were higher for African American mothers when compared to white mothers.

Woodruff et al. [32] using data from the EPA Ambient Air Monitoring observed that Hispanic, African-American, and Asian/Pacific Islander mothers experienced higher mean levels of air pollution and were more than twice as likely to live in the most air polluted counties across the country compared with White mothers. In addition, there was an increase in the odds of preterm delivery (*AOR* = 1.05; 95% CI, 0.99–1.12) in a county with high air pollution.

Le et al. [33] reported a stronger association between air pollutants and PTB for Blacks than Whites. PTB was associated with SO_2_ (*OR* 1.07, 1.01–1.14) exposure in the last month of pregnancy, while O_3_ exposures exceeding 92 parts per billion (*OR* 1.08, 1.02–1.14) were associated with PTB in the first months of pregnancy.

### Air Pollution and Small for Gestational Age

Of all 16 studies reviewed, six assessed SGA. Benmarhnia et al. [30] explored racial disparities in SGA in California using data provided by the U.S. Environmental Protection Agency (EPA) and the California Air Resources Board (CARB) to assign chronic air pollution exposures to each birth record, linked by maternal zip code of residence. The predicted difference in probability of SGA between Black and White infants was 0.084 (95% CI: 0.081, 0.087). Together, individual demographics, neighborhood socioeconomic environment (such as unemployment and poverty rates), and neighborhood air pollution explained 37.8% of the Black–White disparity. There was a higher prevalence of SGA among non-Hispanic Blacks (18.3%) as compared to non-Hispanic Whites (9.9%).

In a cohort of 457,642 births in North Carolina, using Bayesian measurement of PM_2.5_ and ozone, Gray et al. [27] reported that infants born to NHB mothers and Hispanic mothers were at an increased odds of SGA (*AOR* = 2.18, 95% CI: 2.12 to 2.24 and *AOR* = 1.21, CI: 1.17 to 1.26, respectively), compared to NHW mothers. After controlling for race and individual and area-level socio-economic status, this difference persisted, suggesting that air pollution is an additional contributor to the observed outcomes.

In a retrospective cohort study of 4,098,750 births in California, Woodruff et al. [32] observed that Hispanic, African-American, and Asian/Pacific Islander mothers experienced higher mean levels of air pollution, determined from strategically placed EPA monitors. They were also more than twice as likely to live in the most polluted counties compared with White mothers, after controlling for maternal risk factors, region, and educational status (Hispanic mothers: *AOR* = 4.66; 95% CI: 1.92–11.32; African-American mothers: *AOR* = 2.58; 95% CI: 1.00–6.62; Asian/Pacific Islander mothers: *AOR* = 2.82; 95% CI, 1.07–7.39]. However, there was no significant increase in the odds of SGA (*AOR* = 0.96; 95% CI, 0.86–1.07) in counties with higher air pollution.

In a cohort of 164,905 births in Michigan, Le et al. [33] showed that there was an association between term SGA with exposure to CO and NO_2_ during the first trimester of pregnancy. They also reported an association between term SGA and exposure to O_3_ and PM_10_ during the later stages of pregnancy. There was evidence of stronger associations between CO and term-SGA, NO_2_ and term-SGA, and SO_2_ and term-SGA for infants of Black mothers as compared to White mothers.

Rich et al. [34] found significantly increased risk of SGA associated with first and third trimester exposures to PM_2.5_ and increased risk of very small for gestational age (VSGA) associated with first, second, and third trimester exposures to high NO_2_ concentrations. According to this study, mothers of SGA and VSGA infants were more likely to be less than 25 years old and less likely to have completed high school, compared to mothers of appropriate-size births. They were also more likely to be single, African American, and to have smoked during pregnancy.

### Air Pollution and Low Birth Weight

Low birth weight was assessed in five of the 16 studies reviewed. Gray et al. [27] used multivariate analysis of factors including PM_2.5_ and O_3_ to assess their association with weight differences in grams and 95% CI for all births. PM_2.5_ exposures were associated with LBW among infants born to NHB and Hispanic mothers more than those born to NHW mothers (−187.5 g, 95% CI: −183.6 to −191.4 and −46.8 g, 95% CI: −41.8 to −51.7, respectively).

Miranda et al. [35] characterized maternal exposure to traffic-related air pollution during pregnancy by using residential proximity to major road ways as a proxy. Women residing within 250 m of a major roadway were at 3–5% increased odds of having a LBW baby than women residing more than 250 meters away (*p*<0.05). The mean birth weight was 3376 g for NHW, 2114 g for NHB, and 3330 g for Hispanics.

In a retrospective cohort study in California [36], African Americans, despite being a significantly lower percentage of the study population, had 3.19% of the LBW infants, compared to 1.23% for whites and 1.59% for Hispanics. Bell et al. [37] also reported that the association between air pollutants (especially PM_2.5_) and LBW for infants of Black mothers was stronger than for White mothers.

In a large cohort study of 1,548,904 births in Texas, Geer et al. [38] reported that interquartile increases in ambient air pollutant concentrations of SO_2_ and O_3_ were associated with a 4.99 g (95% CI, 1.87–8.11) and 2.72 g (95% CI, 1.11–4.33) decreases in birth weight, respectively. Lower birth weight was associated with exposure to O_3_ in the first and second trimester, whereas results for other pollutants did not differ significantly by trimester.

In a case-control study, Ghosh et al. [39] observed that women who were exposed to secondhand smoke at home had increased odds of term LBW (*AOR* = 1.36; 95% CI: 0.85, 2.18) compared to unexposed women. Blacks and Hispanic had higher odds of having LBW babies compared to White mothers.

### Air Pollution and Stillbirths

Only one cohort study among reviewed studies assessed stillbirths in relation to air pollution. According to DeFranco et al. [11], high average PM_2.5_ exposure through pregnancy was not associated with a significant increase in stillbirth risk (*AOR* 1.21; 95% CI: 0.96, 1.53). There was also no higher risk of stillbirth associated with exposure in either the first or second trimester. However, exposure to high levels of PM_2.5_ in the third trimester of pregnancy was associated with 42% increased risk of stillbirth, (*AOR* = 1.42; 1.06, 1.91). Stillbirth rates were higher among mothers older than 40 years (11.6 per 1000), NHB mothers (8.6 per 1000), and mothers with lower education level and tobacco use.

## Discussion

The objective of this review was to explore the importance of race/ethnicity as a risk factor for air pollution-related poor pregnancy outcomes.

In recent years, issues of racial disparities and air pollution exposure have received increasing attention in the USA and globally. Trends in air pollution and racial disparities suggest worse exposure in people of color (POC) when compared to non-Hispanic whites (NHW). One of the most comprehensive and informative analysis is that of Liu and colleagues [40] who quantified exposure disparities among racial/ethnic groups (NHW, NHB, Hispanic (any race), non-Hispanic Asian) and by income for multiple spatial units (contiguous United States, states, urban vs. rural areas) and years (1990, 2000, 2010) for carbon monoxide, nitrogen dioxide, ozone, particulate matter with aerodynamic diameter ≤2.5 μm and ≤10 μm (PM2.5 and PM10), and sulfur dioxide. They concluded that for all years and pollutants, the racial/ethnic group with the highest national average exposure was a racial/ethnic minority group (NHB), with a national mean air pollution exposure higher for all three racial/ethnic minorities than for NHW. The degree of these disparities varied by pollutant and state. These findings are consistent with other studies that have explored this topic [13, 32, 41–44].

Similarly, trends in outcomes of pregnancy suggest worse outcomes in POC when compared to NHW. Approximately 700 women die in the USA each year as a result of pregnancy or its complications [45]. Pregnancy-related mortality rates are three times higher in black women than white women and two times higher in American Indian/Alaska natives [46]. POC are more likely to have other factors that contribute to maternal and infant mortality including: teenage pregnancy, preterm birth, low birth weight, late or no prenatal care [46–48]. The factors driving these disparities are multifactorial, mostly social and economic factors — income, transportation, education, access to food, differences in access to healthcare including pre-natal care, and differences in health insurance coverage as well as structural and systemic racism and discrimination [14, 45, 46, 49–51].

This review suggests that air pollution is associated with adverse pregnancy outcomes in the USA and higher rates of adverse outcomes among people of color, than among Whites. These findings are consistent with studies conducted in other countries [13, 14, 52]. There are a multitude of factors other than exposure to air pollution that have also been shown to be associated with poor pregnancy outcomes, including socioeconomic status, level of education, and maternal smoking. These factors also tend to be independently associated with increased exposure to air pollutants and poor pregnancy outcomes [53–55]. However, when stratified by race, the exposures and outcomes tend to be worse in minority groups suggesting racial disparities in both exposures to air pollutants and outcomes of pregnancy. Almost all the studies we reviewed were large retrospective cohort studies; therefore, other factors (socio-economic) at least partially explain the disparities observed in the studies reviewed. We point, however, to evidence from molecular epidemiologic studies that suggest biological mechanisms to explain the effects of air pollution on pregnancy outcomes [56–58]. There is little data to suggest that Black mothers have any greater genetic predisposition to adverse effects of air pollution than Whites [26, 30, 58]. Finally, while studies suggest that PTB is associated with SO_2_, and O_3_ exposure, the effects of such pollutants may in part be due to their roles in exacerbating other disease conditions that are known to cause PTB, e.g., hypertension, diabetes, asthma, and preeclampsia [59, 60]. This is also another area for further research. In fact, Casey and colleagues in a recent study found that power plant retirements were associated with a decrease in the proportion of preterm birth within 5 km (−0.019, 95% CI: −0.031, −0.008) and 5–10 km (−0.015, 95% CI: −0.024, −0.007), controlling for secular trends with mothers living 10–20 km away [61].

Even though none of the studies reviewed explored household air pollution, there is evidence that it also contributes to poor pregnancy outcomes, especially in developing countries where cooking is done by burning of biomass in poorly ventilated homes [12, 62–64]. Several measures can be taken to improve air quality and reduce the negative impacts of air pollution on overall health and on gestational health in particular. At individual and household levels, these measures include avoidance of cooking or heating with biomass fuel indoors, ensuring adequate household ventilation, quitting smoking, use of chimneys, and the use of air filters [65]. At state and national levels, policy measures could include carbon tax and subsidies for alternative energy sources [66]. Lu and colleagues [67] proposed a comprehensive 12-point plan to close the black-white gap in birth outcomes that includes increased pre-conception care to POC, expanding access to healthcare to underserved areas, strengthening father involvement in minority families, closing the education gap, supporting working mothers and families, fighting systemic racism, among others.

A potential shortcoming in our review is the limited number of available published studies. We cannot rule out that many studies on this topic with null results may have remained unpublished. We also could not assess how differences in the methods by which air pollution was captured in different studies may have contributed to the reported results. Potential exposure misclassification is certainly an important possibility to consider when evaluating research in this area of investigation if we are to build a corpus of studies reliable enough to guide changes in practice and policy.

We have reviewed existing evidence that suggests a need for further investigation of the impact of air pollution on pregnancy outcomes and how these vary by race and ethnicity. Despite the limitations noted, we believe that this review of 16, US-based studies increases our understanding of the impact of air pollution on maternal-child health and of disparities in maternal-child health across races and ethnicities. With the goal of enhancing the literature and filling gaps in knowledge on this topic, on the basis of our review, we suggest future studies that explore the biologic factors underlying air pollution’s adverse effects; incorporate measurement of household air pollution and personalized exposure measurement; establish the causal mechanisms of air pollution’s impact on specific outcomes, including prematurity, birthweight, IUGR, and fetal deaths; determine the period during pregnancy in which women are most likely to be affected by air pollutants and to establish the specific effects of different pollutants and pollutant mixtures; and explore differential effects of specific air pollutants on Blacks versus Whites.

## Data Availability

Not applicable
